# Measuring malaria morbidity in an area of seasonal transmission: Pyrogenic parasitemia thresholds based on a 20-year follow-up study

**DOI:** 10.1371/journal.pone.0217903

**Published:** 2019-06-27

**Authors:** Marion Dollat, Cheikh Talla, Cheikh Sokhna, Fatoumata Diene Sarr, Jean-François Trape, Vincent Richard

**Affiliations:** 1 Unité d'Epidémiologie des Maladies Infectieuses, Institut Pasteur de Dakar, Dakar, Sénégal; 2 Service de Maladies Infectieuses et Tropicales, Hôpital Avicenne, Assistance Publique-Hôpitaux de Paris (AP-HP), Paris, France; 3 Laboratoire de Paludologie, Institut de Recherche pour le Développement, Dakar, Sénégal; Swiss Tropical & Public Health Institute, SWITZERLAND

## Abstract

**Introduction:**

Asymptomatic carriage of *P*. *falciparum* is frequent in areas endemic for malaria and individual diagnosis of clinical malaria attacks is still difficult. We investigated the impact of changes in malaria endemicity on the diagnostic criteria for malaria attacks in an area of seasonal malaria transmission.

**Methods:**

We analyzed the longitudinal data collected over 20 years from a daily survey of all inhabitants of Ndiop, a rural community in central Senegal, in a logistic regression model to investigate the relationship between the level of *Plasmodium falciparum* parasitemia and the risk of fever, with the aim of determining the best parasitemia thresholds for attributing to malaria a fever episode.

**Results:**

A total of 34,136 observations recorded from July 1993 to December 2013 from 850 individuals aged from 1 day to 87 years were included. *P*. *falciparum* asymptomatic carriage declined from 36% to 1% between 1993 and 2013. A total of 9,819 fever episodes were associated with a positive blood film for *P*. *falciparum*. Using age-dependent parasitemia thresholds for attributing to malaria a fever episode, we recorded 6,006 malaria attacks during the study period. Parasitemia thresholds seemed to be lower during the low-to-zero transmission season and tended to decrease with changes in control policies. The number of clinical malaria attacks was overestimated for all age groups throughout the study when all fever episodes associated with *P*. *falciparum* parasitemia were defined as malaria attacks.

**Conclusion:**

Pyrogenic thresholds are particularly sensitive to changes in malaria epidemiology and are therefore an interesting tool to accurately assess the burden of malaria in the context of declining transmission.

## Introduction

In recent years, the scale-up of new treatments and effective prevention tools has led to major advances in the fight against malaria [1,2]. However, it is still difficult to precisely assess the population-scale impact of these various strategies on malaria morbidity, due to the lack of reliable surveillance data, the varying diagnosis criteria, and the limited epidemiological methods used to estimate the disease burden. Indeed, most individuals in areas endemic for malaria progressively acquire partial and labile immunity, which allows them to tolerate low to moderate levels of parasitemia without experiencing clinical symptoms [3]. Thus, the detection of parasites in the blood film from a febrile individual is not sufficient to distinguish a malaria attack from other causes of fever.

The measurement of parasite density has long been the cornerstone of the approaches to assess malaria morbidity in research and clinical trials in endemic areas [4,5]. Methods based on parasite density were developed in the early 1990s to estimate the fraction of fever cases attributable to malaria in a population [6–8]. Several studies provided evidence for an age-dependent threshold effect in the relationship between the level of parasitemia and the occurrence of fever at the individual level and showed that such pyrogenic parasitemia thresholds can be used to confirm or rule out the diagnosis of clinical malaria attack in a given area and population [7,9–11].

The level of endemicity has also been shown to critically influence the pyrogenic parasitemia thresholds [12,13]. In the current context of declining malaria in many parts of the world, changes in the acquisition of immunity and thus in the resulting levels of parasitemia associated with malaria attacks are expected. Previous studies in Dielmo, an area with intense and perennial malaria transmission in Senegal, have shown that the supervised introduction of combination therapy for first-line treatment of malaria attacks and the deployment of long-lasting insecticide-treated nets (LLINs) were associated with a dramatic decrease in parasite density levels in asymptomatic individuals and altered pyrogenic thresholds for *P*. *falciparum* malaria attacks in all age-groups [13]. Here, we analyze the longitudinal data collected uninterruptedly during a 20-year period in the neighboring community of Ndiop, Senegal, an area with seasonal malaria transmission typical of most Sahelo-Sudanian savannah areas of West Africa. Our objectives were to investigate morbidity evolution according to transmission decrease, and to determine the trend of pyrogenic parasitemia thresholds for diagnosing *P*. *falciparum* malaria attack on the road towards malaria elimination.

## Population and methods

### Study area

The study was performed in Ndiop, a village located in central Senegal approximately 290 km southeast of Dakar and 10 km from the Gambian border (15°95’N, 16°35’W). The area is characterized by an average annual rainfall of 750 millimeters, concentrated during the rainy season between June and October, followed by a dry season of seven to eight months.

A prospective longitudinal study was carried out from July 1993 to December 2013 to investigate the relationship between malaria host, vector, and parasite. Both active and passive detection of fever cases was performed and regular cross-sectional surveys of malaria prevalence conducted.

### Procedures

The procedures of medical, parasitological, entomological, and epidemiological surveillance were the same as those used in the village of Dielmo and have already been described [13,14].

Briefly, inclusion in the cohort was offered to all villagers at the beginning of the study, and to any newcomer thereafter. During twenty years from July 1993 to December 2013, all households included in the study were visited daily except Sundays and nominative information on the inhabitants, including the presence of fever or other symptoms (allegation of fever, asthenia, headache, vomiting, diarrhea, abdominal pain, cough) were recorded. Blood tests were performed for all suspected or confirmed cases of fever. Asymptomatic malaria carriage was investigated by performing cross-sectional surveys for all included individuals at regular intervals: every week during the first six months of the study, then every month unless rare exceptions, and finally three times a year from 2004 onwards: at the beginning and end of the dry season (January or February, and May or June), and at the end of the rainy season (October or November). Blood was taken by finger stick and 200 oil-immersion fields were examined. The parasite/leukocyte ratio for each plasmodial species was measured. For readability and clinical relevance reasons, parasitemia was expressed in trophozoites/μl: to overcome the lack of simultaneous measurement of leukocytemia, we adopted a mean standard leukocyte count of 8,000 per μl of blood for all age groups, following WHO recommendations and in accordance with data from a preliminary investigation in the study area [15,16].

Four first-line drug regimens were successively used for malaria attack treatment during the study period: oral quinine (July 1993 to December 1994), chloroquine (January 1995 to October 2003), sulfadoxine-pyrimethamine plus amodiaquine (SP+AQ: November 2003 to May 2006), and artesunate plus amodiaquine (Artemisinin-based combination therapy or ACT: from June 2006). Until 2011, antimalarial drugs were systematically given to young children (less than 5 years old) with fever associated with a parasitemia ≥ 2400 trophozoites/μl, whereas the choice to treat or not with antimalarial drugs was left to the appreciation of the physician or nurse when parasitemia was lower. Only symptomatic treatment was generally given to adults and older children permanently living in the village (except for pregnant women). This attitude was justified by the willing to limit parasitic resistance to antimalarials at that time, and by the fact that a number of fever cases with positive parasitemia did not necessarily correspond to malaria, in a context where the close surveillance of the villagers allowed immediate reactivity in case of clinical worsening. The treatment policy was modified in 2011 to limit malaria transmission and since then, ACT has been systematically given to all febrile patients showing the presence of trophozoites in blood films, independent of age and parasite density.

LLINs were deployed in Ndiop in July 2008, at the time of their distribution throughout the country. Malaria transmission averaged 43 infected bites per person per year before the introduction of combination therapy, with high annual variations (maximum 169 in 2001), and steeply decreased to very low levels after the deployment of LLINs (average of five for the period from 2008 to 2013) (personal data).

### Parasite prevalence

Parasite prevalence was measured during cross-sectional studies in all villagers enrolled in the project. Comparisons of the prevalence rate between seasons were analyzed using the Fisher exact test to investigate seasonal variations.

### Definition of case and control observations

Febrile cases were identified by active and passive surveillance and compared with asymptomatic controls, identified during cross-sectional investigations. We included only individuals who had participated in at least four consecutive cross-sectional surveys in the period from 1993 to 1997 in the analysis, when these studies were performed monthly, or at least three surveys in one year during the subsequent years. We excluded observations performed during pregnancy and those showing the presence of two or more parasitic species. We established a baseline during the first six months of follow-up to have a reference period, keeping only observations performed before the first antimalarial intake for each individual. Definitions of case and control were the same as those used for the previous work about the Dielmo cohort [13]. Briefly, fever cases corresponded to observations for which rectal temperature was ≥ 38°C or axillary temperature ≥ 37.5°C, and control observations were those recorded during cross-sectional surveys with rectal/axillary temperature < 38°C/37.5°C and no episode of illness within 15 days before and seven days after the thick smear was performed.

### Pyrogenic threshold calculations

The same previous method for pyrogenic threshold calculations applied to the Dielmo cohort was used [13]. Risk of fever was analyzed using logistic regression of age and *P*. *falciparum* parasitemia. Odds ratio estimated by the model could be used to measure the association between risk of fever and parasitemia variation. Parasitemia could affect the risk of fever as a continuous variable and also as a binary one: the existence of a threshold effect had been previously demonstrated [9,13]. Our data suggested a variation of the threshold level according to age: following the method applied to Dielmo cohort, we aimed to estimate this pyrogenic threshold in order to attribute a fever episode to malaria. To define precisely the shape and the model of this age-dependent threshold, we define it as a function of age and five parameters *(a*, *b*, *c*, *d*, *e)* whose values could be estimated by successive fits, as previously described [9,13]. The section below describes the steps of modeling leading us to estimate these parameters, and so the pyrogenic thresholds.

At first, bivariate analysis was performed to explore the association between risk of fever, parasitemia and age. Comparing several age groups with each other, the best fit based on deviance criterion was obtained using a series of *k* = 5 dummy variables for the following age groups: 0–23 months, 24–59 months, 5–9 years, 10–14 years, and ≥15 years. Previous studies had shown that parasitemia thresholds for attributing fever episodes to malaria decreased in relation to control policies and decrease in transmission [12,13]. The treatment period (i.e., baseline, quinine, chloroquine, SP+AQ, ACT, and ACT+LLINs periods) was therefore expected to affect the relationship between parasite density and fever, due to the impact on the reservoir and the changes in malaria transmission. This was further supported by the model: considering the probability of fever associated with parasitemia and treatment period in bivariate analysis, stratification on the treatment period revealed significantly different ORs. This effect modifier was also found in the logistic regression model. Consequently, we separately analyzed the six treatment periods. We increased the power of analysis by pooling the two shortest periods (SP+AQ and ACT), which had a similar profile, in a global period called “Bitherapy”. The January to June semester was defined as the low-to-zero transmission season and the July to December semester as the high transmission season based on entomological and climatic data (unpublished). Since Ndiop is a seasonal transmission area, the relationship between fever and parasitemia may differ from one season to another. Using the same method used for treatment period (ie stratification in bivariate analysis then integration of interaction between the effect of age and that of parasitemia in the logistic regression model), we found that the season significantly impacted the relationship between parasitemia and fever, which led us to analyze our data for each season separately. At each modeling step an individual random effect was tested quantifying unmeasured or unmeasurable inter-individual variability. Akaike criterion (AIC) was used to compare models with and without random effect [17]. Analyses were performed using the lme4 package of R software version 3.4 [18,19].

As previously shown, the logit of the probability πij that the individual i presents a fever episode during the observation j can be expressed as a linear function of age *z*_*ik*_ (with *k* representing the five age-groups) and parasitemia *x*_*ij*_ (model A):

$$logit(\pi_{ij})=\beta_{0}+\sum_{k=1}^{5}\beta_{1k}\;z_{ik}+\beta_{2}f(x_{ij})+\alpha_{i}$$ (model A)

In this model, *β*_0_ was a constant, *β*_1_ and *β*_2_ the regression coefficients, and *α*_*i*_ the random-effects individual term. Looking for the best manner to describe the fever risk *f*(*x*_*ij*_) as a continuous function of parasitemia, different functions were tested: linear, log and power of x (parasitemia). The best criterion of model selection (deviance) was obtained for the *r*th power function of parasitemia. The parsimonious exponent *r* was then tested and obtained based on the model deviance for each treatment period and within each treatment period for both transmission seasons for different values with a precision of 0.01 (S1 and S2 Figs).

The existence of a threshold effect, in addition to the previous continuous effect of parasitemia, was previously demonstrated [9,13]. It was introduced as a binary variable *s*_*ij*_ in the model (model B):

$$logit(\pi_{ij})=\beta_{0}+\sum_{k=1}^{5}\beta_{1k}\;z_{ik}+\beta_{2}{\left(x_{ij}\right)}^{r}+\beta_{3}s_{ij}+\alpha_{i}$$ (model B)

*s*_*ij*_ took the value 0 when the *j*th parasitemia of the individual *i* was below the tested threshold and the value 1 when it was higher. We tested constant age-independent thresholds at different values and then age-dependent thresholds. LOESS (locally weighted smoothing) was used in regression analysis to describe the relationship between age and parasitemia (S1 and S2 Figs). The trend of the loess curve could be approximated by two different equations whose shape depends on five parameters (*a*, *b*, *c*, *d*, *e*) as previously described [9,13]. We identified *a* as age of maximum parasitemia, *b* as the highest parasitemia, *c* as the parasitemia at age 0, *d* as the level of parasitemia in the oldest adults, and *e* as the shape of the decrease. The first equation (*h*_1_) concerned the youngest children before age *a* (age of maximal parasitemia), whereas the second (*h*_2_) was applied to older children and adults after age *a*:

$$h_{1}(z_{i})=[z_{i}(2a)-z_{i}{}^{2}][(b-c)/a^{2}]+c$$

$$h_{2}(z_{i})=\{[a(2a)-a^{2}][(b-c)/a^{2}]+c–d\}\{exp[-e(z_{i}-a)]\}+d$$

By varying these five parameters *(a*, *b*, *c*, *d*, *e)* on a defined set, we obtained many combinations enabling us to define a binary threshold variable. This variable was coded in 1 or 0 according to whether the parasite density was below or above the curve of h1 and h2, and introduced into the model B as a qualitative variable. All models were compared with each other using the AIC criterion. For each study period, the model with the smallest AIC was retained with the five corresponding estimated parameters [17].

### Definition of *P*. *falciparum* malaria attack

A *P*. *falciparum* clinical malaria attack was defined as any case with fever or fever-related symptoms for which parasitemia was higher than the threshold derived from the above model. Cases were counted separately if they occurred 15 days or more apart.

### Comparison of malaria attack definitions

We then tested four parasitemia definitions and compared the number of malaria attacks determined by each: (A) episode of illness (fever or fever-related symptoms) with parasitemia higher than the age-dependent threshold measured for the corresponding season and treatment period; (B) episode of illness with parasitemia higher than the age-dependent threshold measured for the corresponding treatment period, regardless of the season; (C) episode of illness with parasitemia higher than the constant threshold of 5,000 trophozoites/μl, frequently used in malaria endemic areas; and (D) episode of illness associated with the presence of malaria parasites, regardless of the level of parasitemia.

### Ethical considerations

The project was initially approved by the Ministry of Health of Senegal and the assembled village population. Approval was then renewed on a yearly basis with written informed consent from individuals enrolled in the project and the parents or guardians of the children enrolled. The National Ethics Committee of Senegal and *ad-hoc* committees of the Ministry of Health, The Pasteur Institutes (Dakar and Paris), and the Institut de Recherche pour le Développement (IRD) regularly performed audits.

## Results

### *P*. *falciparum* prevalence

We measured parasite prevalence in 41,334 blood films collected during 159 cross-sectional surveys from all present villagers, irrespective of clinical symptoms. Before the beginning of the project, a preliminary survey carried out in June 1993 (end of the dry season) showed a parasite prevalence of 17%. We observed that prevalence reached 39% on average during the quinine period (which includes two rainy seasons and one dry season) and remained high during the chloroquine period. It started to decrease after the beginning of the combination therapy period (average prevalence of 14% between November 2003 and July 2008), and fell further after the deployment of LLINs (1%). There were consistently wide variations between seasons: parasite prevalence was significantly lower during the low-to-zero malaria transmission season. This difference was found for each treatment period, except at the last years of the study upon the distribution of LLINs (Table 1).

**Table 1:** *P*. *falciparum* prevalence rate (sexual forms and gametocytes) during cross-sectional surveys in Ndiop according to season and treatment period.

	Prevalence rate during high transmission season (%)	Prevalence rate during low-to-zero transmission season (%)	P value*
**Quinine** **(07/1993-12/1994)**	41.5	26.5	<0.001
**Chloroquine** **(01/1995-10/2003)**	23.5	16	<0.001
**Bitherapy** **(11/2003-07/2008)**	19.9	10.2	<0.001
**ACT+LLINs** **(08/2008-12/2013)**	1.6	1.3	0.41

* *p* values were calculated using the Fisher exact test

### Pyrogenic thresholds

We excluded 176 individuals (453 observations) because of insufficient follow up, 1,246 observations performed during pregnancy, and 3,201 observations with a thick blood film positive for two or more parasitic species when determining the parasitemia thresholds used to define a *P*. *falciparum* attack. A total of 34,136 observations recorded from July 1993 to December 2013 from 850 individuals aged from 1 day to 87 years were included: 22,827 observations matched the definition for a case of fever and 11,309 for that of a control observation.

### *P*. *falciparum* parasitemia during fever episodes

**Fig 1:**
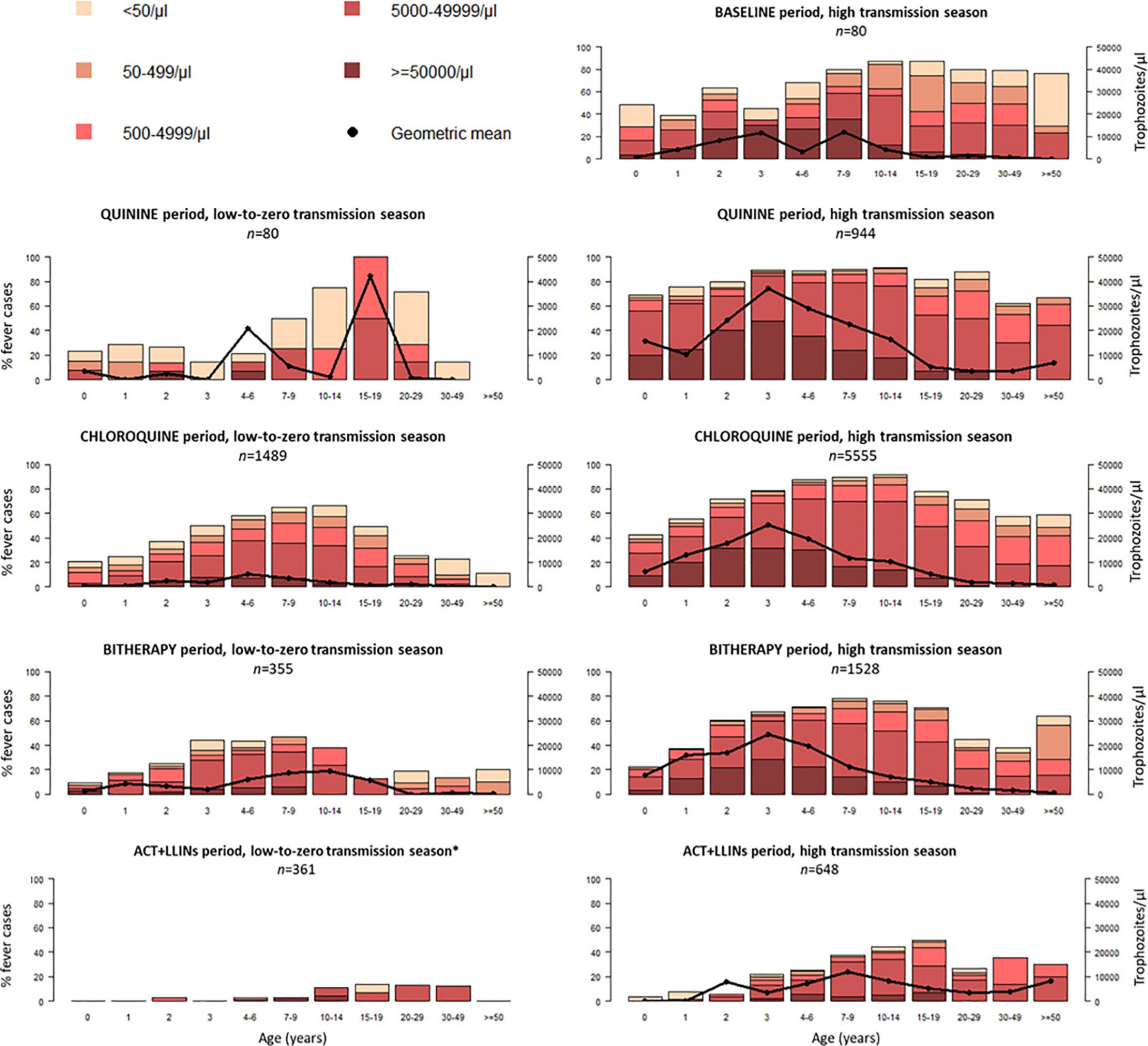
Age distribution of parasite prevalence rate, class of parasite density, and mean *P*. *falciparum* parasitemia observed during all causes of fever episodes for each study period. *Geometric mean incalculable.

The proportion of fever cases harboring *P*. *falciparum* trophozoites and the mean level of parasitemia during fever episodes decreased markedly during the 20 years of the study (Fig 1). Parasite prevalence was lower in children below three years of age than in older children and adults, regardless of the treatment period. The highest levels of parasitemia were observed in three-year-old children, except at the end of the study, in which children between seven and nine years old had the highest levels. During the 1993 high malaria transmission season, 71% of thick blood films from febrile patients were positive for *P*. *falciparum*. This proportion remained high during the quinine and chloroquine periods (84% and 77%, respectively), then decreased during the bitherapy period (62%) and more so after the introduction of LLINs (26%). During the low-to-zero malaria transmission season, the prevalence of fever associated with positive parasitemia was lower: 32%, 44%, and 28% during the quinine, chloroquine, and bitherapy periods respectively, and 4% in years following introduction of LLINs.

### Asymptomatic *P*. *falciparum* infections

**Fig 2:**
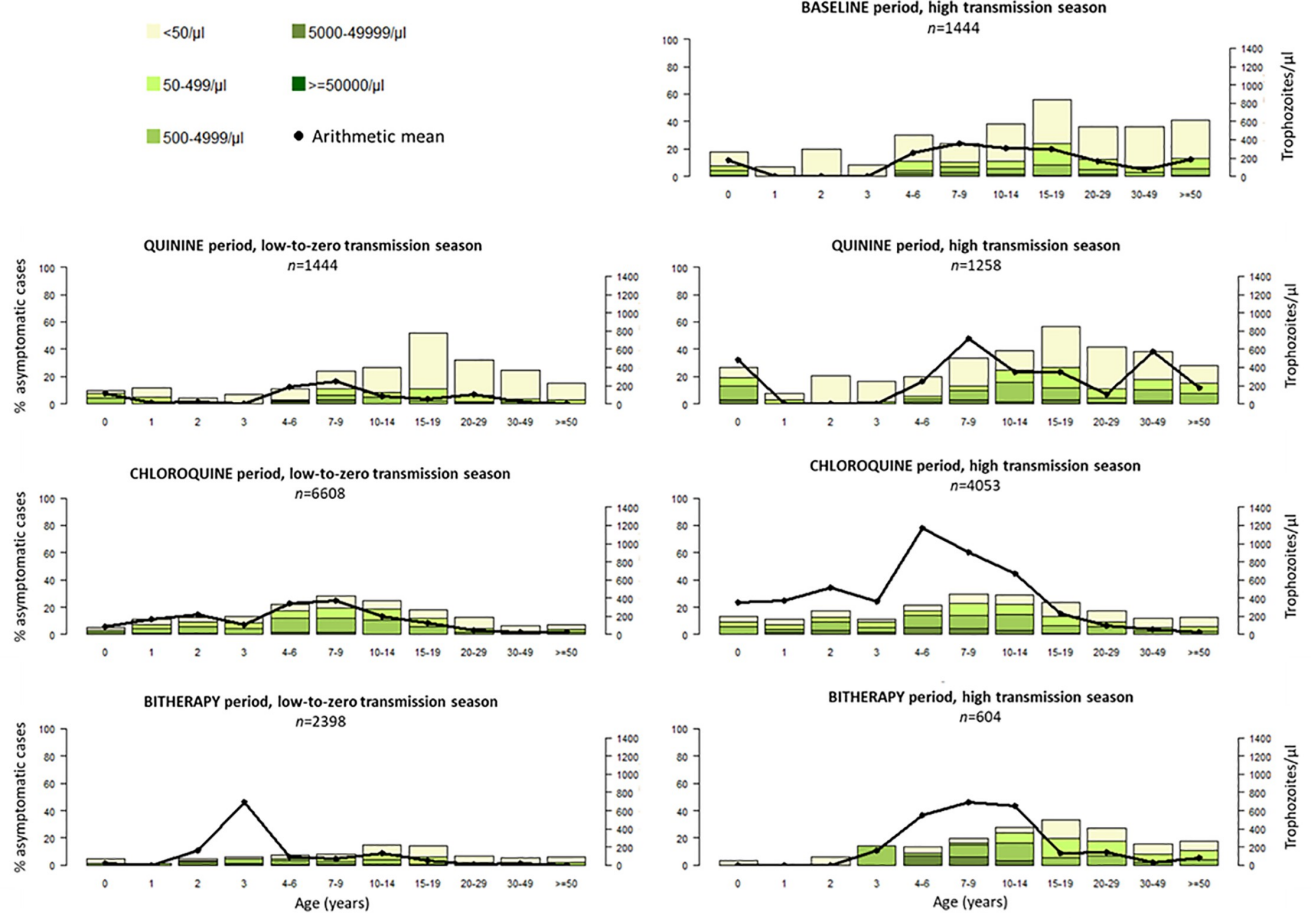
Age distribution of parasite prevalence rate, class of parasite density, and mean asymptomatic *P*. *falciparum* parasitemia in control observations for each study period. Data for the ACT+LLINs period are not displayed because levels were too low.

**Fig 3:**
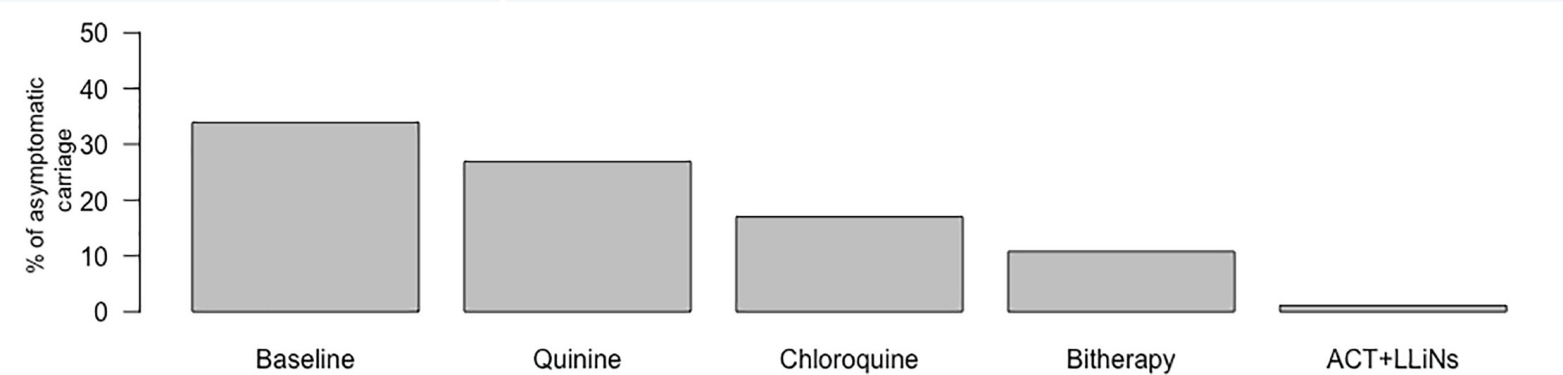
Changes in asymptomatic carriage, Ndiop, July 1993—December 2013.

Parasite prevalence and the mean level of parasitemia during asymptomatic infections are shown for each period, season, and age group in Fig 2. At the beginning of the study, asymptomatic carriage concerned 36% of the Ndiop villagers, with a maximal prevalence of 56% among young adults (15–19 years). The proportion in the whole population gradually decreased afterwards during the quinine (27%), chloroquine (17%), and bitherapy (118%) periods. Asymptomatic carriage was very low (1%) during the most recent period (Fig 3). We observed the highest asymptomatic parasitemia levels in children between 4 and 10 years old, the highest mean being 1,200 trophozoites/μl during the chloroquine period in the rainy season for the four to six-year age group. Asymptomatic carriage was lower during the low-to-zero transmission season during the first 15 years of the study: 22% of villagers were asymptomatic carriers in the low-to-zero transmission season *vs* 32% in the high transmission season during the quinine period, 16% *vs* 19% during the chloroquine period, and 8% *vs* 20% during the bitherapy period. Asymptomatic carriage further declined after the introduction of LLINs and was low in both seasons: 1% during the low-to-zero transmission season *vs* 1% during the high transmission season.

### Attributing fever episodes to *P*. *falciparum* malaria

**Fig 4:**
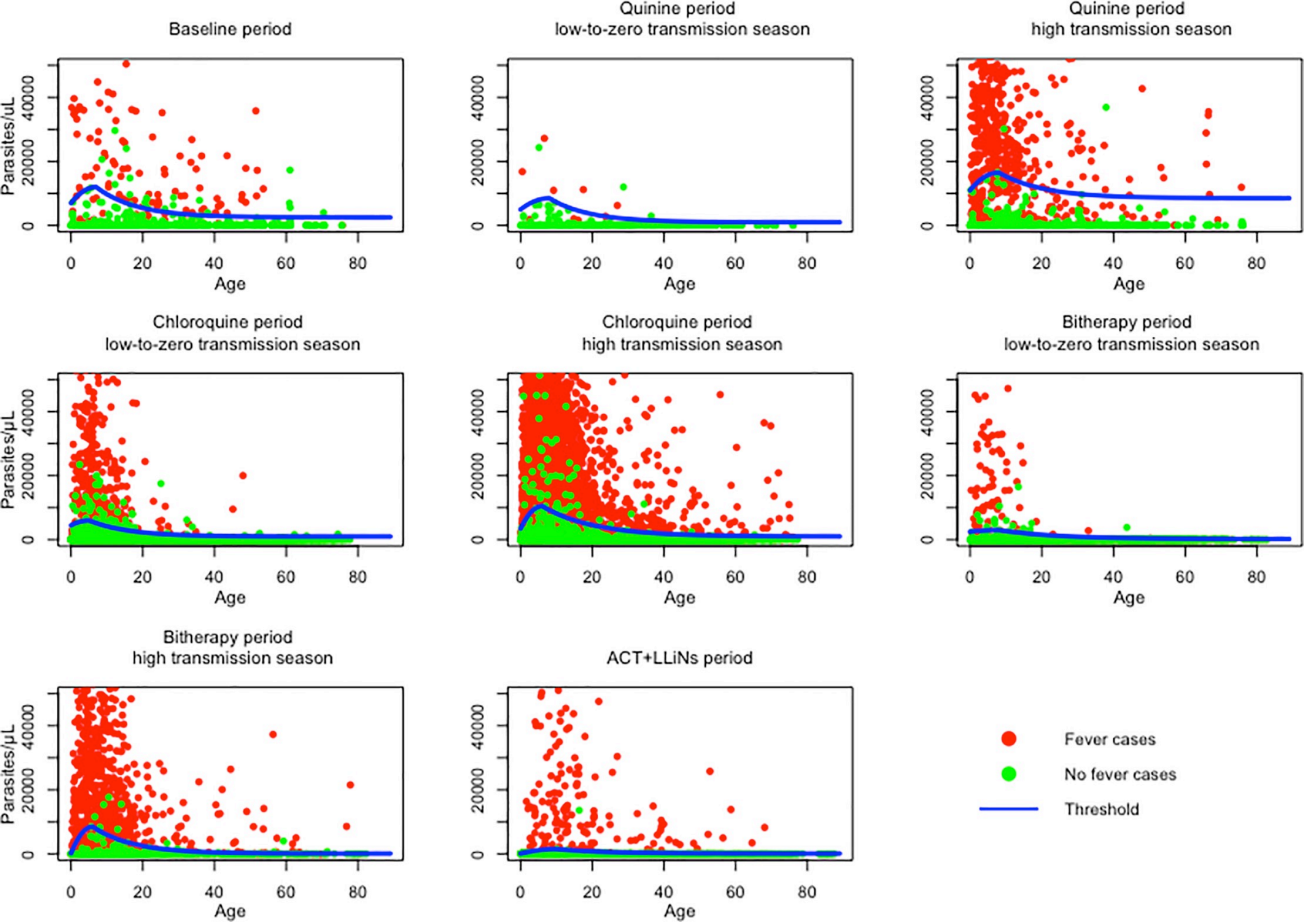
Parasite density by age, season, and period of treatment, with pyrogenic threshold determined by the model.

Our data showed the existence of two distinct groups based on parasite density, which suggested a possible existence of a parasitemia threshold between fever and no fever cases (Fig 4). For each treatment period and within each treatment period for both transmission seasons, we tested this threshold effect by introducing the binary variable *s*_*ij*_ in the model: we found a significant association for each study period. Parameters based on the lowest deviance that define the shape and level of pyrogenic thresholds during the baseline and each season for the four treatment periods by age are given in Table 2.

**Table 2:** Estimates of the parameters defining the age-dependent pyrogenic threshold according to season for each study period.

	Baseline period	Quinine period, Low-to-zero transmission season	Quinine period, high transmission season	Chloroquine period, Low-to-zero transmission season	Chloroquine period, high transmission season	Bitherapy period, Low-to-zero transmission season	Bitherapy period, high transmission season	ACT+LLINs period
**Number of observations**	3007	1524	2202	8097	9608	2753	2132	4813
**Number of individuals**	327	312	333	580	590	453	418	472
**Total number of case observations/case observations with positive parasitemia†**	349/248 (71.1%)	80/26 (32.5%)	944/792 (83.9%)	1489/659 (44.3%)	5555/4295 (77.3%)	355/100 (28.2%)	1528/953 (62.4%)	1009/183 (18.1%)
**Total number of control observations/case observations with positive parasitemia†**	2658/957 (36.0%)	1444/322 (22.3%)	1258/405 (32.2%)	6608/1056 (16.0%)	4053/754 (18.6%)	2398/203 (8.5%)	604/122 (20.2%)	3804/41 (1.1%)
**Exponent for the function of parasitemia *(r)***	0.26	0.35	0.42	0.53	0.28	0.41	0.42	0.26
**Age of maximum parasitemia *(a)*** **††**	7	8	8	5	6	8	6	10
**Maximum parasitemia threshold *(b)*** **†††**	12 000	8500	16500	6000	10500	3000	8500	1500
**Parasitemia threshold at year 0 *(c)*** **†††**	7000	5000	11000	4500	3500	2500	200	100
**Lowest parasitemia threshold in adults *(d)*** **†††**	2500	1000	6500	1000	1000	200	100	100
**Shape of the decrease *(e)***	0.09	0.09	0.05	0.09	0.07	0.06	0.09	0.07
**Threshold effect OR (95% CI)**	3.36** (1.57–7.18)	23.19** (2.63–261.59)	6.05* (1.61–39.59)	2.66*** (1.56–4.56)	2.27*** (1.58–3.29)	5.01* (1.44–17.69)	2.55* (1.10–6.20)	27.49** (3.46–606.8)
**Continuous effect of parasitemia OR (95% CI)**	1.34*** (1.27–1.43)	1.05 (0.98–1.12)	1.08*** (1.07–1.09)	1.02*** (1.02–1.02)	1.31*** (1.28–1.33)	1.05*** (1.02–1.08)	1.06*** (1.04–1.08)	1.45*** (1.24–1.70)
**0–23 months OR (95% CI)**	1	1	1	1	1	1	1	1
**24–59 months OR (95% CI)**	0.69 (0.41–1.14)	0.73 (0.38–1.40)	0.71 (0.46–1.12)	0.49*** (0.40–0.59)	0.59*** (0.48–0.72)	0.56** (0.39–0.79)	0.72 (0.45–1.16)	0.76* (0.60–0.95)
**5–9 years OR (95% CI)**	0.47** (0.28–0.80)	0.22*** (0.10–0.47)	0.52** (0.34–0.81)	0.23*** (0.18–0.28)	0.33*** (0.27–0.40)	0.19*** (0.13–0.28)	0.37*** (0.24–0.55)	0.44*** (0.35–0.56)
**10–14 years OR (95% CI)**	0.15*** (0.08–0.28)	0.13*** (0.04–0.36)	0.32*** (0.19–0.53)	0.15*** (0.12–0.19)	0.20*** (0.16–0.25)	0.12*** (0.07–0.19)	0.21*** (0.13–0.32)	0.34*** (0.26–0.45)
**≥15 years OR (95% CI)**	0.18*** (0.12–0.27)	0.12*** (0.06–0.24)	0.44*** (0.30–0.66)	0.10*** (0.09–0.12)	0.18*** (0.15–0.21)	0.11*** (0.07–0.15)	0.14*** (0.10–0.21)	0.20*** (0.16–0.25)

Abbreviations: ACT, artemisinin-based combination therapy; LLINs, long-lasting-insecticide-treated nets; CI, confidence interval; OR, odds ratio.

**†** *P*. *falciparum*, asexual forms

**††** age in years

**†††** parasitemia in trophozoites/μL.

* p<0.05

** p<0.01

*** p<0.001

Modeling failed to identify a specific threshold for the low-to-zero transmission season of the ACT+LLINs period, due to the small number of observations with positive parasitemia (38 observations) such that the relationship between parasitemia and risk of fever was no longer significant. There was thus a single threshold for the ACT+LLINs period, independent of the season.

**Fig 5:**
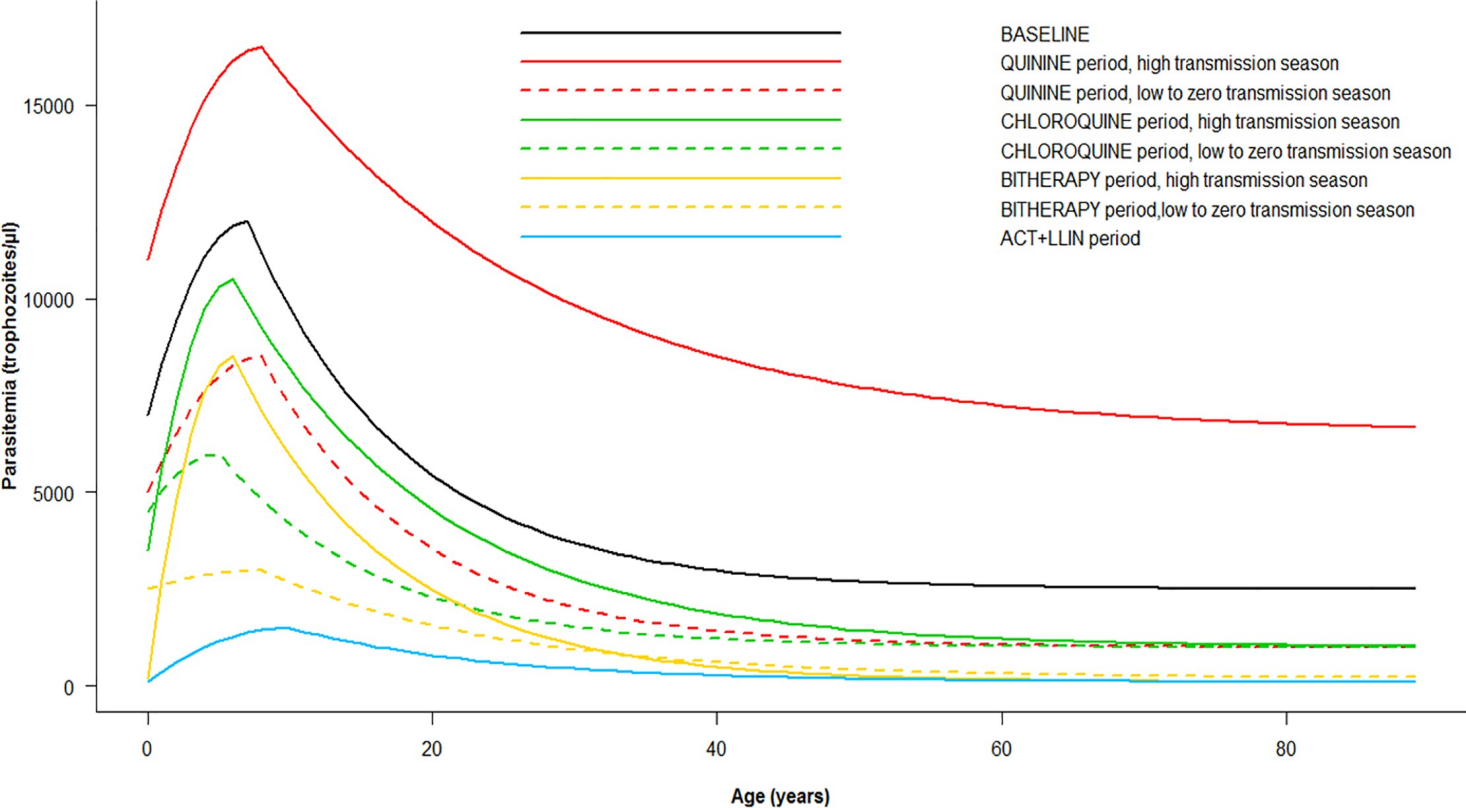
Random-effect logistic regression model-derived threshold levels of *P*. *falciparum* parasitemia for attributing fever episodes to *P*. *falciparum* malaria by age, season, and period of treatment.

The Fig 5 presents the age-dependent thresholds across the periods. The level of thresholds tended to decrease from the first periods to the ACT+LLINs period. The highest thresholds were observed among children between 5 and 8 years old, except in the last years of the study (ACT+LLINs period) during which the highest threshold shifted to older children. The thresholds seemed to be higher during the high transmission season than the low-to-zero transmission season especially among children and young adults.

We compared the four definitions of clinical malaria attack. Considering definition A as the reference (episode of illness with parasitemia higher than the age-dependent threshold measured for the corresponding season and treatment period), definition D (episode of illness with positive parasitemia, whatever its level) considerably overestimated the number of clinical malaria attacks for all age groups and all periods (Table 3). During the course of the study, a total of 9,819 fever episodes were associated with a positive blood film for *P*. *falciparum*, within which 6,006 could be attributed to malaria using the age-dependent parasitemia thresholds. Overestimation was greatest during the quinine and chloroquine periods during the low-to-zero transmission season (264% and 138%, respectively), and lowest during the ACT+LLINs period, regardless of the season (25% and 21%). When a constant threshold of 5,000 trophozoites/μl was used (definition C), the number of clinical malaria attacks for the quinine and chloroquine periods was underestimated during the low-to-zero transmission season (-9% and -6%, respectively), but overestimated during the high transmission season (+19% and +5%, respectively), with disparities depending on age. This threshold led to an underestimation of the number of attacks for almost all age groups for the bitherapy period, regardless of the season. Finally, the use of a season-independent threshold led to a substantial overestimation of malaria attacks for the quinine and chloroquine periods (7% and 10%, respectively).

**Table 3:** Number of *P*. *falciparum* malaria attacks by age, season, and treatment period, according to four definitions of malaria attacks.

Period	Malaria definitions	Age (in years)								Total (children)	Total (adults)	Total
		[0–1]	[2–3]	[4–6]	[7–9]	[10–14]	[15–19]	[20–29]	[≥ 30]	[0–14]	[≥15]	All ages
**Baseline**	**A**	**28**	**33**	**43**	**30**	**29**	**21**	**28**	**41**	**163**	**90**	**253**
	**B**	NA	NA	NA	NA	NA	NA	NA	NA	NA	NA	NA
	**C**	29 (+3.6%)	33 (+0.0%)	45 (+4.7%)	33 (+10.0%)	31 (+6.9%)	22 (+4.8%)	27 (-3.6%)	36 (-12.2%)	171 (+4.9%)	85 (-5.6%)	256 (+1.2%)
	**D**	47 (+67.9%)	45 (+36.4%)	60 (+39.5%)	45 (+50%)	50 (+72.4%)	53 (+152.4%)	68 (+142.9%)	95 (+131.7%)	247 (+51.5%)	216 (+140%)	463 (+83.0%)
**Quinine period (low-to-zero**	**A**	**1**	**1**	**2**	**2**	**0**	**1**	**3**	**1**	**6**	**5**	**11**
**transmission season)**	**B**	1 (-0.0%)	1 (-0.0%)	2 (-0.0%)	0 (-100%)	0 (-0.0%)	1 (-0.0%)	2 (-33.3%)	1 (-0.0%)	4 (-33.3%)	4 (-20%)	8 (-27.3%)
	**C**	1 (-0.0%)	1 (-0.0%)	2 (-0.0%)	2 (-0.0%)	0 (-0.0%)	1 (-0.0%)	2 (-33.3%)	1 (-0.0%)	6 (-0.0%)	4 (-20%)	10 (-9.1%)
	**D**	6 (+500%)	5 (+400%)	3 (+50%)	3 (+50%)	5 (+∞%)	4 (+300%)	11 (+266.7%)	3 (+200%)	22 (+266.7%)	18 (+260%)	40 (+263.6%)
**Quinine period (high**	**A**	**59**	**102**	**154**	**104**	**79**	**20**	**16**	**17**	**498**	**53**	**551**
**transmission season)**	**B**	59 (-0.0%)	102 (+0.0%)	154 (+0.0%)	104 (+0.0%)	82 (+3.8%)	25 (+25%)	27 (+68.7%)	38 (+123.5%)	501 (+0.6%)	90 (+69.8%)	591 (+7.3%)
	**C**	70 (+18.6%)	114 (+11.8%)	171 (+11.0%)	126 (+21.2%)	96 (+21.5%)	27 (+35%)	27 (+68.7%)	25 (+47.1%)	577 (+15.9%)	79 (+49.1%)	656 (+19.1%)
	**D**	93 (+57.6%)	131 (+28.4%)	198 (+28.6%)	147 (+41.3%)	129 (+63.3%)	60 (+200%)	57 (+256.2%)	80 (+252.9%)	698 (+40.2%)	197 (+271.7%)	895 (+62.4%)
**Chloroquine period**	**A**	**26**	**73**	**131**	**77**	**92**	**31**	**13**	**14**	**399**	**58**	**457**
**(low-to-zero transmission**	**B**	26 (-0.0%)	73 (-0.0%)	131 (-0.0%)	76 (-1.3%)	91 (-1.1%)	30 (-3.2%)	13 (-0.0%)	14 (-0.0%)	397 (-0.5%)	57 (-1.7%)	454 (-0.7%)
**season)**	**C**	26 (-0.0%)	76 (+4.1%)	133 (+1.5%)	75 (-2.6%)	86 (-6.5%)	20 (-35.5%)	9 (-30.8%)	6 (-57.1%)	396 (-0.8%)	35 (-39.7%)	431 (-5.7%)
	**D**	113 (+334.6%)	163 (+123.3%)	250 (+90.8%)	166 (+115.6%)	205 (+122.8%)	83 (+167.7%)	43 (+246.2%)	65 (+364.3%)	897 (+124.8%)	191 (+229.3%)	1088 (+138.1%)
**Chloroquine period**	**A**	**273**	**509**	**777**	**576**	**664**	**298**	**166**	**216**	**2799**	**680**	**3479**
**(high transmission**	**B**	281 (+2.9%)	538 (+5.7%)	845 (+8.8%)	660 (+14.6%)	763 (+14.9%)	342 (+14.8%)	204 (+22.9%)	238 (+10.2%)	3087 (+10.3%)	784 (+15.3%)	3871 (+11.3%)
**season)**	**C**	281 (+2.9%)	549 (+7.9%)	856 (+10.2%)	659 (+14.4%)	726 (+9.3%)	301 (+1.0%)	153 (-7.8%)	124 (-3.7%)	3071 (+9.7%)	578 (-15.0%)	3649 (+4.9%)
	**D**	403 (+47.6%)	664 (+30.5%)	1049 (+35.0%)	861 (+49.5%)	1063 (+60.1%)	550 (+84.6%)	393 (+136.7%)	516 (+138.9%)	4040 (+44.3%)	1459 (+114.6%)	5499 (+58.1%)
**Bitherapy period (low-to-zero**	**A**	**10**	**15**	**25**	**15**	**22**	**6**	**2**	**5**	**87**	**13**	**100**
**transmission season)**	**B**	10 (-10.0%)	13 (-13.3%)	22 (-12.0%)	13 (-13.3%)	17 (-22.7%)	4 (-33.3%)	1 (-50.0%)	5 (-0.0%)	75 (-13.8%)	10 (-23.1%)	85 (-15.0%)
	**C**	10 (-10.0%)	13 (-13.3%)	23 (-8.0%)	13 (-13.3%)	17 (-22.7%)	3 (-50.0%)	1 (-50.0%)	1 (-80.0%)	75 (-13.8%)	5 (-61.5%)	80 (-20.0%)
	**D**	16 (+60.0%)	26 (+73.3%)	33 (+32.0%)	23 (+53.3%)	29 (+31.8%)	15 (+150%)	9 (+350%)	15 (+200%)	127 (+46.0%)	39 (+200%)	166 (+66.6%)
**Bitherapy period(high**	**A**	**69**	**122**	**218**	**176**	**168**	**79**	**49**	**79**	**753**	**207**	**960**
**transmission season)**	**B**	68 (-1.4%)	121 (-0.8%)	218 (-0.0%)	175 (-0.6%)	161 (-4.2%)	76 (-3.8%)	48 (-2.0%)	74 (-6.3%)	743 (-1.3%)	198 (-4.3%)	941 (-2.0%)
	**C**	63 (-8.7%)	125 (+2.4%)	240 (+10.1%)	187 (+6.2%)	166 (-1.2%)	70 (-11.4%)	35 (-28.6%)	30 (-62.0%)	781 (+3.7%)	135 (-34.8%)	916 (-4.6%)
	**D**	100 (+44.9%)	157 (+28.7%)	290 (+33.0%)	267 (+51.7%)	261 (+55.4%)	138 (+74.7%)	87 (+77.6%)	131 (+65.8%)	1075 (+42.8%)	356 (+72.0%)	1431 (+49.1%)
**ACT+LLINs period**	**A**	NA	NA	NA	NA	NA	NA	NA	NA	NA	NA	NA
**(low-to-zero transmission**	**B**	**0**	**1**	**1**	**1**	**5**	**1**	**2**	**1**	**8**	**4**	**12**
**season)**	**C**	0 (-0.0%)	0 (-100%)	1 (-0.0%)	1 (-0.0%)	5 (-0.0%)	1 (-0.0%)	2 (-0.0%)	1 (-0.0%)	7 (-12.5%)	4 (-0.0%)	11 (-8.3)
	**D**	0 (-0.0%)	1 (-0.0%)	2 (+100.0%)	1 (-0.0%)	6 (+16.7%)	2 (+100.0%)	2 (-0.0%)	1 (-0.0%)	10 (+25.0%)	5 (+25.0%)	15 (+25.0%)
**ACT+LLINs period**	**A**	NA	NA	NA	NA	NA	NA	NA	NA	NA	NA	NA
**(high transmission**	**B**	**4**	**12**	**18**	**21**	**38**	**33**	**22**	**35**	**93**	**90**	**183**
**season)**	**C**	4 (-0.0%)	9 (-25%)	15 (-16.7%)	20 (-4.8%)	34 (-10.5%)	20 (-39.4%)	15 (-31.8%)	15 (-57.1%)	82 (-11.8%)	50 (-44.4%)	132 (-27.9%)
	**D**	13 (+225%)	14 (+16.7%)	23 (+27.8%)	24 (+14.3%)	43 (+13.2%)	40 (+21.2%)	30 (+36.4%)	35 (+0.0%)	117 (+25.8%)	105 (+16.7%)	222 (+21.3%)

A = episode of illness with parasitemia higher than the age-dependent threshold measured for the corresponding season and treatment period

B = episode of illness with parasitemia higher than the age-dependent threshold measured for the corresponding treatment period, regardless of the season

C = episode of illness with parasitemia higher than the constant threshold of 5,000 trophozoites/μl, frequently used in malaria endemic areas

D = all episodes of illness associated with the presence of malaria parasites, regardless of the level of parasitemia

## Discussion

In this cohort of villagers living in an initially moderate and seasonal malaria transmission area, we observed that malaria epidemiology and the pyrogenic parasitemia thresholds guiding the definition of malaria attacks tend to change over time. These results corroborate those observed in Dielmo, a neighboring locality with initial intense and perennial malaria transmission and where the same treatment policies and control measures were implemented in parallel [13,19].

Parasite prevalence progressively decreased with the implementation of new malaria control strategies. It decreased in the early years of the study, possibly due to the project itself, which resulted in more administered treatments. Thereafter, it decreased when combination therapy replaced chloroquine as first-line treatment. Finally, the most important decline followed the deployment of LLINs: parasite prevalence fell to 1% by the end of the study, and gametocyte carriage to 0.2%. These changes mimic those observed in Dielmo, where mean *P*. *falciparum* prevalence was 69% during the first year of the study in 1990 (*vs* 26% during the first full year of study in Ndiop), and fell to 0.3% in 2012 [19].

Asymptomatic carriage was more frequent during the first two periods than during subsequent periods, but parasite densities were lower: high prevalence of low density carriers in all age groups could reflect robust immunity acquired before the beginning of the survey. During the study, the peak prevalence of fever with presence of trophozoites on blood film (regardless of the level of parasitemia) shifted gradually towards older children and young adults. In correlation with the dramatic decline observed in asymptomatic carriage, this result highlights the fact that reduced exposure consecutive to malaria control intensification is reflected in waning immunity in adults. It illustrates that was observed in serological studies [20].

The pyrogenic parasitemia thresholds seemed to vary substantially during the study period and to be highest during the high transmission season in all periods except the last years, although the method used here do not allow us to demonstrate a significant difference between periods and between seasons. Pyrogenic threshold is an old concept in malariology [21]. It involves a discontinuous relationship between fever and parasitemia, of which the existence was confirmed under holoendemic perennial malaria conditions in the 1990s with the development of efficient statistical methods [9]. Our results show the existence of an age-dependent threshold effect of parasitemia on the risk of fever in a mesoendemic malaria setting with seasonal transmission, but at lower levels for all age group than in holoendemic area as in Dielmo [13] (S3 Fig). Thresholds were higher in children aged 5 to 10 years than in younger children for all periods, unlike in Dielmo, where the highest levels were observed in children aged 1 to 2 years [13]. This difference could be explained by a slower acquisition of immunity, due to lower cumulated exposure to infected Anopheles bites, as the level of the parasite threshold reflects the balance between the parasite and the individuals’ immune response.

Malaria control strategies seem to modify pyrogenic parasitemia threshold levels, as previously observed in Dielmo [13]. The only exception concerned the quinine period, where the pyrogenic threshold determined for the high transmission season was higher than that determined for the baseline. Several hypotheses can be put forward. At first, the short duration of the quinine period (18 months) make its analysis particularly sensitive to interannual variations. Secondly, the baseline period has been built keeping only the first treated fever episode for each individual: subsequent episodes, with higher parasitaemia, were recorded in the quinine period. Thus, the threshold levels at baseline may be artificially low.

Pyrogenic threshold levels varied depending on the season: they tended to be lower during the low-to-zero transmission season. Similar results have been reported in areas of similar endemicity in several studies with various approaches to determine the parasitemia thresholds used for case definition of malaria attack [10,11,22,23]. The decrease in threshold levels during the dry season may be linked to a rapid decrease in anti-parasitic immunity following the interruption of transmission. Indeed, some studies have shown a rapid decrease in antibody levels against *P*. *falciparum* during the dry season in areas of seasonal malaria transmission [24–26].

Our study shows the persistence of a pyrogenic threshold during the ACT+LLINs period, although malaria became hypoendemic and asymptomatic carriage quite rare. This may reflect, in part, the long-term persistance of some immunity as observed in immigrants exposed to malaria after years or even decades without contact with the parasite [27]. The role of memory B cells and long-term persistence of IgG antibody-secreting cells has been proposed [27,28]. It also appears that there is a persistent antigenic stimulus that is undetectable by microscopy, requiring more sensitive tools, such as PCR, to be measured. However, a recent study in Ndiop indicated quite unfrequent sub-microscopic asymptomatic carriage in 2013 [20]. Thus, the role of such sub-microscopic infections in maintaining immunity is yet to be demonstrated [29].

Our results confirm that parasite density is a key determinant of malaria morbidity in endemic areas with seasonal transmission. Defining malaria attacks as all fever cases associated with any level of parasitemia overestimated the malaria burden for all age groups in an endemic area. This was observed during all study periods and remained true during the last years of the study, although to a lesser degree, when malaria became hypoendemic.

This study has several limitations. First, it is well known that there are rapid changes in parasite density during the same fever episode [30]. Following the methodology previously applied to the Dielmo data, we considered in the analysis only the highest level of parasitaemia when several thick blood smears were performed during the same episode to reduce this measurement bias, but we are aware that this assumption might tend to push the pyrogenic threshold up. However, since this criterion of highest parasitaemia was applied throughout the study, it does not prevent us from studying the evolution of the thresholds according to the treatment and prevention strategy. Second, the existence of genetic factors in the immune response to the parasite, as well health of individual, micro-heterogeneity in transmission, and recent exposure, is likely to result in variability of the threshold between individuals. Nevertheless, for each period, the five parameters *a*, *b*, *c*, *d*, and *e* estimated by the model remained unchanged whether the random-effect was included or excluded.It must also be acknowledged that the choice to group data by periods did not take into account dynamic changes within treatment periods, such as variations observed in annual parasite prevalence, wear and tear of the nets, or changing in clinicians appreciation to treat or not with antimalarial drugs a fever case with low parasitemia. However, implementation of new strategies at the village level seemed to be the most relevant breaking point for study epidemiological changes. The thresholds determined for Ndiop may not be extrapolated to other settings with similar endemicity: malaria transmission is spatially heterogeneous and a recent study showed the complexity of such spatial distribution in eight neighboring villages of Ndiop [31]. Finally, asymptomatic carriage may have been underestimated particularly in the last years of the study, first because optical microscopy used here is less sensitive than molecular biology tools and second because it was estimated three times a year during the 10 last years of the study rather than monthly at the beginning.

## Conclusion

The pyrogenic parasitemia threshold model is applicable to cohort studies in the context of seasonal malaria transmission, as previously documented for intense and perennial transmission settings. Pyrogenic thresholds are not fixed and particularly sensitive to the evolution of the epidemiological profile, and are therefore an interesting tool to accurately assess the burden of malaria in the context of declining transmission.

## Supporting information

S1 Fig A: Parasite density by age (loess curves in solid red line), B: zoom of A on low values.
(TIF)

S2 Fig Fitting of regression model by *r*. The parameter *r* is the exponent of the power function of parasite density used for modeling the relationship between parasitemia and fever risk as a continuous function.
(TIF)

S3 Fig Comparison of parasitemia thresholds for attributing fever episodes to *P. falciparum* malaria between the villages of Ndiop and Dielmo, by period of treatment. The thresholds presented for Ndiop are those of the high transmission season.
(TIF)
