# Supplementary figures and images for: Measuring malaria morbidity in an area of seasonal transmission: Pyrogenic parasitemia thresholds based on a 20-year follow-up study
Source: PLoS One. 2019 Jun 27;14(6):e0217903. doi: 10.1371/journal.pone.0217903 (PMC6597048; doi:10.1371/journal.pone.0217903)

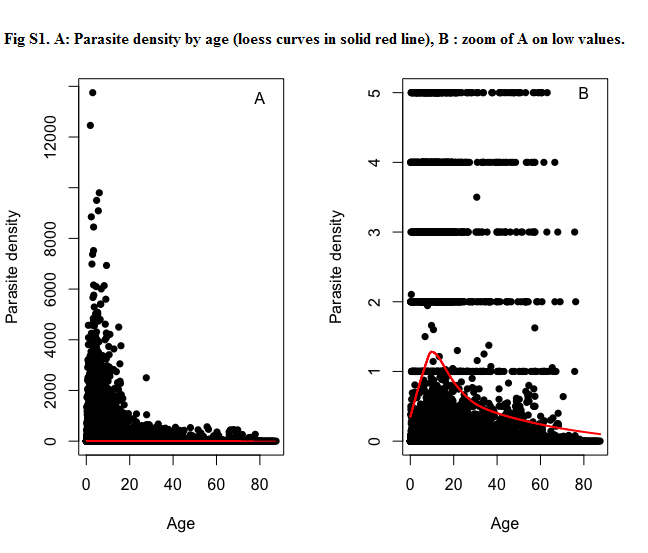

Supplement: S1 Fig — A: Parasite density by age (loess curves in solid red line), B: zoom of A on low values. (TIF) [file pone.0217903.s001.tif]

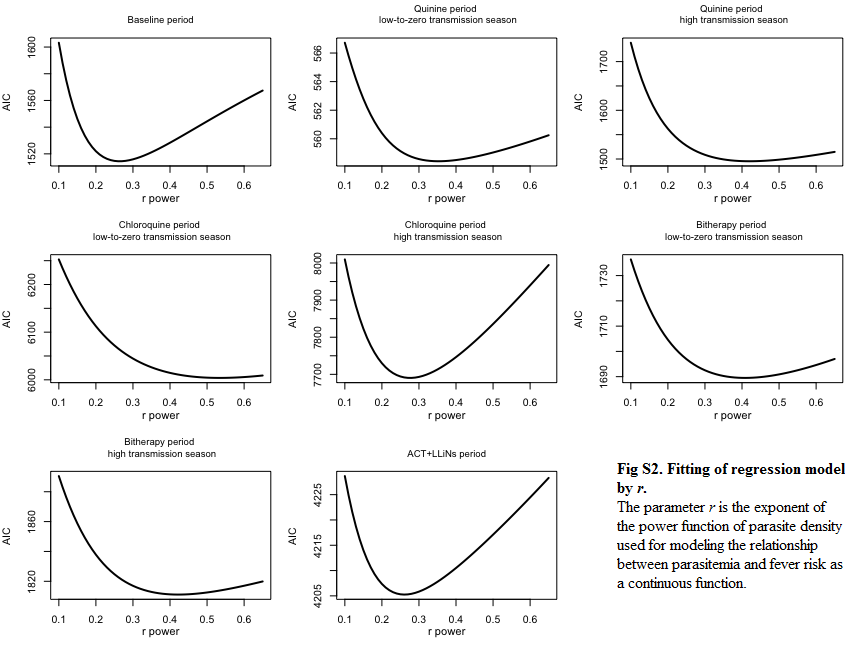

Supplement: S2 Fig — The parameter r is the exponent of the power function of parasite density used for modeling the relationship between parasitemia and fever risk as a continuous function. (TIF) [file pone.0217903.s002.tif]

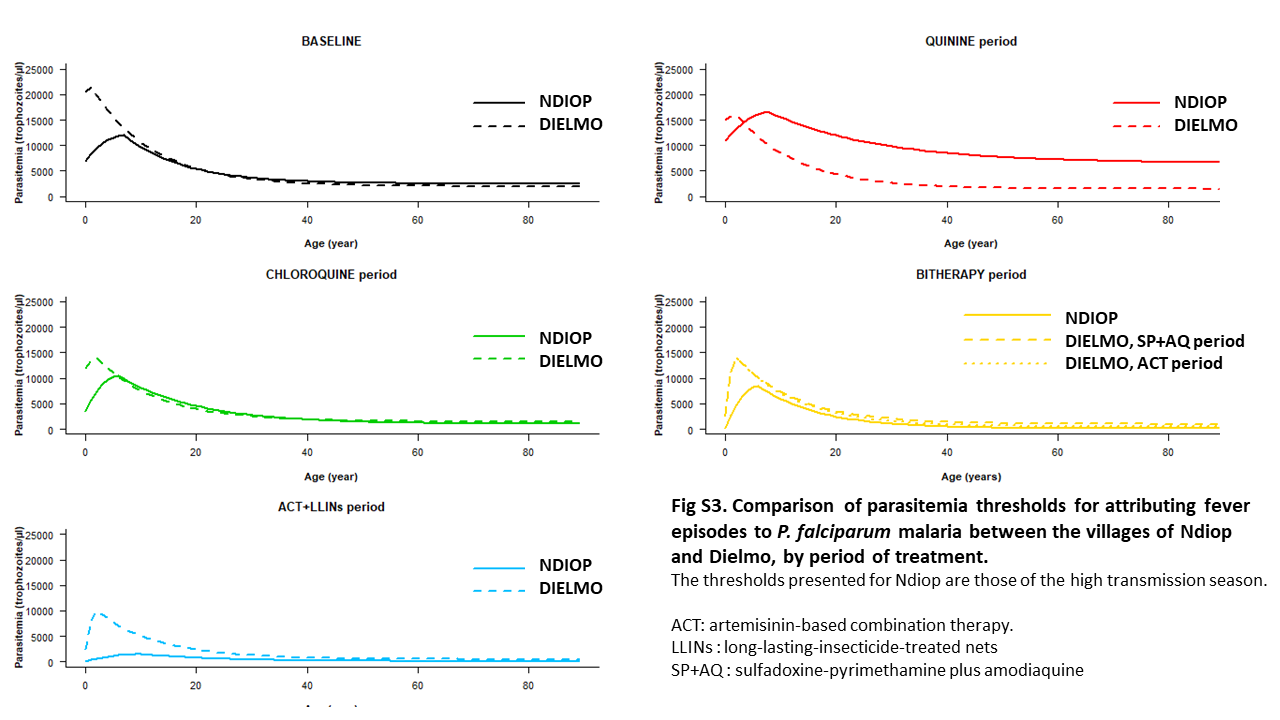

Supplement: S3 Fig — The thresholds presented for Ndiop are those of the high transmission season. (TIF) [file pone.0217903.s003.tif]
